# Wall Fabrication by Direct Energy Deposition (DED) Combining Mild Steel (ER70) and Stainless Steel (SS 316L): Microstructure and Mechanical Properties

**DOI:** 10.3390/ma15175828

**Published:** 2022-08-24

**Authors:** Virginia Uralde, Alfredo Suarez, Eider Aldalur, Fernando Veiga, Tomas Ballesteros

**Affiliations:** 1 Department of Engineering, Public University of Navarre, Los Pinos Building, Campus Arrosadía, 31006 Pamplona, Spain; 2 TECNALIA, Basque Research and Technology Alliance (BRTA), Parque Científico, Parque Científico y Tecnológico de Gipuzkoa, 20009 Donostia-San Sebastián, Spain; 3 ADDILAN Fabricación Aditiva S.L., Eguzkitza 1, 48200 Durango, Spain

**Keywords:** wire arc additive manufacturing, SS 316L, mild steel, microstructure, additive manufacturing

## Abstract

Direct energy deposition is gaining much visibility in research as one of the most adaptable additive manufacturing technologies for industry due to its ease of application and high deposition rates. The possibility of combining these materials to obtain parts with variable mechanical properties is an important task to be studied. The combination of two types of steel, mild steel ER70-6 and stainless steel SS 316L, for the fabrication of a wall by direct energy deposition was studied for this paper. The separate fabrication of these two materials was studied for the microstructurally flawless fabrication of bimetallic walls. As a result of the application of superimposed and overlapped strategies, two walls were fabricated and the microstructure, mechanical properties and hardness of the resulting walls are analyzed. The walls obtained with both strategies present dissimilar regions; the hardness where the most present material is ER70-6 is around 380 HV, and for SS 316L, it is around 180 HV. The average values of ultimate tensile strength (UTS) are 869 and 628 MPa, yield strength (YS) are 584 and 389 MPa and elongation at break are 20% and 36%, respectively, in the cases where we have more ER70-6 in the sample than SS 316L. This indicates an important relationship between the distribution of the materials and their mechanical behavior.

## 1. Introduction

Research on additive manufacturing (AM) technologies and their various possibilities has increased in the last decade. Most applications have been developed by studying samples and parts made from a single material. The benefits have been enormous, demonstrating the potential of these technologies to replace traditional subtractive manufacturing technologies. Currently, research is being carried out that is looking into the possibility of combining different materials for the manufacture of a single part. Bimetallic structures (BMSs) can be applied when two different zones present specific property requirements. This helps maximize performance in every area of the component. BMSs combine metals such as steels, nickel-based materials, aluminum or titanium, and they find their application in aerospace, naval and automotive industries. When expensive high-performance alloys need to be employed, BMSs allow us to reduce the cost of the part by using this material only in the specific location where it is needed [1,2]. Serving this purpose, wire arc additive manufacturing (WAAM) provides acceptable results, as it achieves higher material deposition rates, making it more productive and, therefore, more cost-effective. Another peculiarity of this technology is its applicability to all weldable metals, thus allowing the use of a wide range of materials at a reduced cost [3].

Bimetallic structures can be made by different material combinations. A suitable couple of materials must be chosen in order to avoid defects. In effect, chemical and physical properties must be considered, as they cannot always be corrected by adjusting the process parameters. Different Coefficients of Thermal Expansion (CTEs) can lead to stresses, especially when cooling or if any thermal treatment is applied, because of different contraction behavior. Chemical incompatibility of the materials to be used can lead to the formation of brittle or unstable interfaces in the matrix [4]. The migration of elements (including Fe, Cr, Ni, Mn, W, Mo and Nb) from one region to another can determine the properties of the joining and the final part. The appearance or not of each compound determines the formation of secondary phases [5,6]. The dispersion of elements between the two alloys can be depicted. The hardness and the microstructure of each region, as well as the variation on the interface, are also studied.

Hereafter, some common material combinations and their benefits are mentioned. Copper–stainless steel (SS) which combines the high conductivity of copper with the high corrosion resistance of stainless steel. The main applications are for fusion reactors, heat exchangers or cookware [7]. The high corrosion resistance of the aluminum makes aluminum–steel bimetallic parts to be employed as adapters on oxygen regenerators or as details in equipment for electrolytic refining [8]. High-quality Nickel–Steel combinations are difficult to obtain due to the different material properties. Despite this, by means of this combination of materials excellent mechanical properties, good corrosion and radiation damage resistance and high-temperature creep strength are obtained, being suitable for nuclear applications or gas turbines [2]. The Inconel is a nickel-based superalloy. Inconel718-austenitic SS combination has been employed in the aeronautic sector and in some nuclear power plants [9,10]. Regarding steels, different types of them can be combined in function of the desired properties, for example, low carbon steel with austenitic stainless steel [3], maraging steel with tool steel [11], ferritic steel with austenitic steel [12] or martensitic with austenitic stainless steels [13]. M. Ahsan et al. [14] fabricated a BMS from low-carbon steel (LCS) and austenitic stainless steel and then heat-treated it. This methodology improved the ultimate tensile strength (UTS) data, the yield strength (YS) and the elongation by 25%, 35% and 250%, respectively. This improvement was due to the transformation of the ferritic microstructure of the as-deposited LCS into ferrite–bainite.

The main objective of this work was to test the additive manufacturing by direct energy deposition of walls composed of two combined materials. The paper shows the necessary conditions for the fabrication of stainless-steel and mild steel walls by means of a superimposition and overlapping strategy. The novel approach of this research was to create mild steel and stainless-steel walls fabricated by WAAM and to study their mechanical properties, hardness and microstructure. These guidelines are intended to serve as a starting point for the fabrication of real parts with a combination of weldable materials capable of being deposited by means of WAAM technology.

## 2. Materials and Methods

In the work presented in this paper, two different materials, ER70S-6 steel and stainless steel SS 316L, were used, always in the form of wires measuring 1.2 mm in diameter. The composition of these materials is shown in Table 1. The substrate used was a flat plate of SS 316L with a thickness of 10 mm.

**Figure 1:**
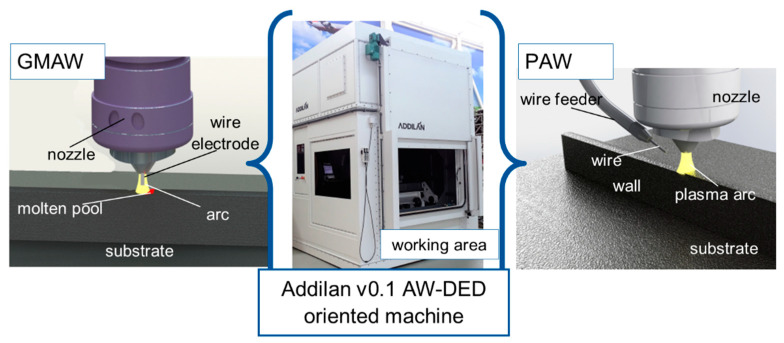
ADDILAN v0.1 machine configurations for additive manufacturing with GMAW and PAW technologies.

The samples were manufactured on the ADDILAN V0.1 machine (see Figure 1), which has a working volume of 1300 mm × 900 mm × 500 mm for weights up to three hundred kilograms. It has a dedicated numerical control that allows access to variables and process parameters. Among the processes and technologies to be integrated into the machine, WAAM is available, mainly Plasma Arc Welding (PAW), Gas Tungsten Arc Welding (GTAW) or Gas Metal Arc Welding (GMAW), with interchangeable torches, depending on the material and metallic properties desired. These torches are integrated into a 4-axis head. In addition, it has an inert chamber in which there is a tilting table with Cartesian coordinates.

In this work, the selected technologies are PAW for the material stainless steel 316L-Si. For the ER70-6 material, GMAW was used. In PAW, a Tetrix 552 AC/DC synergic plasma arc welding system was used, and in GMAW, a Titan XQ 400 AC pulse welding equipment was used, both from EWM AG (Mündersbach Germany).

In this case, the table was left fixed for the manufacture of straight walls. The stainless-steel 316L substrate was strapped to the table and subsequently cleaned to begin the deposition process. The length of the walls was fixed at 220 mm, and the beads were applied, layer by layer, up to a sufficient height for the extraction of tensile mechanical test specimens.

Table 2 provides a general description of the manufacturing conditions used for both materials. ER70S-6 mild steel uses GMAW technology with pulsed MAG welding mode. Ar gas (80%) + CO_2_ (20%) at a flow rate of 17 L/min was chosen as protection gas, and a nozzle measuring 20 mm in diameter was chosen. In SS 316L stainless steel, PAW technology was used with a 100% Ar protection gas at a flow rate of 15 L/min. For correct deposition, a height control was used in which a setpoint voltage was entered and the machine adjusted the height to maintain a constant voltage.

In both technologies, an Optris CT pyrometer (Berlin, Germany) was used to measure the temperature of the deposited material during and between layers. In addition, a Rapidox R2100 oxygen level analyzer from the manufacturer Cambridge-Sensotec (St. Ives, United Kingdom) was used.

The conditions in Table 2 were selected on the basis of a preliminary study carried out. The selection of parameters involved designing a battery of tests to find the optimal variables of the wall manufacturing process. In this battery of tests, unit strands were manufactured, and the width and height of the wall were measured. The aim was to characterize, based on the input variables (intensity and voltage of the heat source, travel speed and feed speed), the optimum manufacturing conditions of the layer (width, height and continuity). The methodology for carrying out this preliminary study is presented in the paper by the authors Aldalur et al. [15].

**Figure 2:**
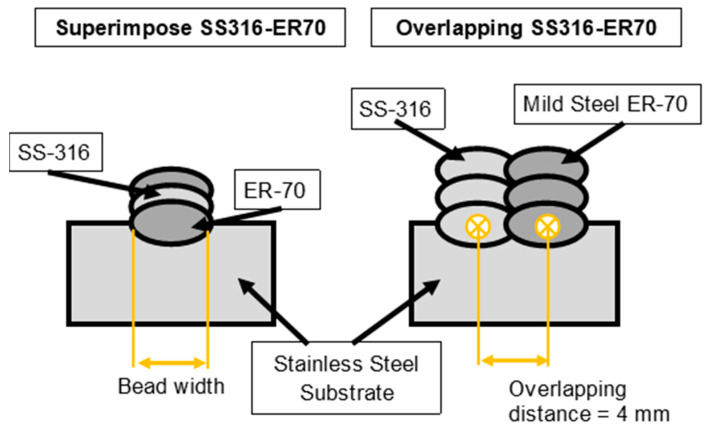
Two-dimensional illustration with dimensions of features for both strategies.

Figure 2 describes the position of both materials in an illustrative way, as well as presenting the arrangement of the different materials and the fundamental distances taken in the process.

To carry out the mechanical characterization tests, the ISO 6892-1 standard [16] was followed. Specimens were removed from the fabricated walls as deposited. The test direction of the specimen is the forward direction of the torch. Four tensile test specimens were purchased in total. Two for each different wall. The terminals of the test specimens have a metric 6 thread. Mechanical tests at room temperature were performed at a speed of 1 mm/min, using an Instron 5585H machine (Norwood, MA, USA) equipped with a load cell (100 kN). In this work, metallographic samples were extracted by conventional machining of the inner surface of additive manufactured walls. The samples were then mechanically polished and etched with a 2% solution of Nital—nitric acid and ethanol—to reveal the grain structure. Finally, microhardness tests were carried out on the two fabricated walls. In the different regions corresponding to the deposition of each of the materials. The microhardness measurement was carried out by using an EMCOTEST DuraScan G5 microhardness tester (Kuchl, Austria), applying a load of 98 N (10 kgf).

**Figure 3:**
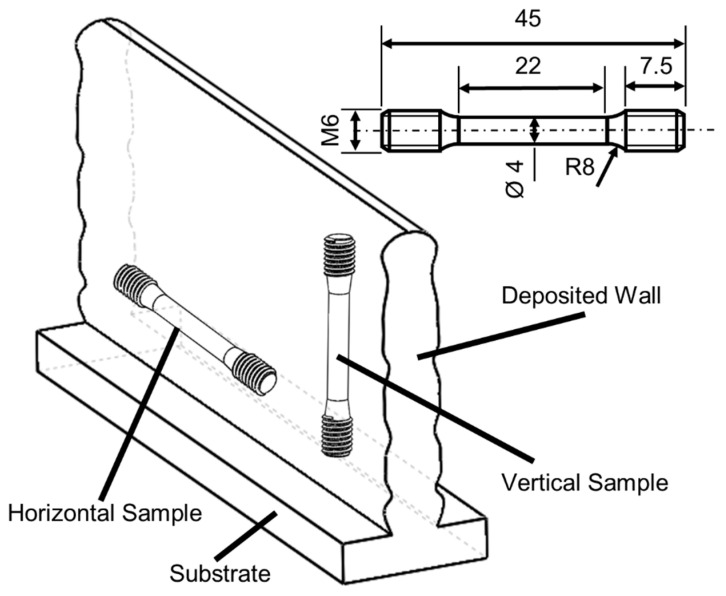
Layout and distribution of specimens with engineering drawing for tensile test.

Figure 3 shows the arrangement of the samples and the industrial drawing corresponding to the standard. In the preliminary tests, 6 specimens were tested in each direction in the case of ER70 and 4 specimens in the case of SS 316L. For the bimetallic wall specimens, 4 specimens were tested in the horizontal direction. The horizontal direction corresponds to the direction of torch advancement and the vertical direction to the direction of wall growth. For the manufacture of the specimens, a rectangle of the specimen is removed by saw cutting, and the roughing, finishing and thread cutting are carried out on a numerically controlled lathe. The aim is to analyze the influence of grain anisotropy and the existence of interfaces in the different layers due to the heating–cooling phases on the mechanical properties.

## 3. Results

### 3.1. Preliminary Tests

In this section, we explain the preliminary tests on both materials for the fine-tuning of the process and the results of mechanical tests and microstructure analysis.

#### 3.1.1. Mild Steel GMAW–WAAM

A preliminary parameter selection study to find the optimal parameters for the wire arc additive manufacturing of mild steel material was carried out. Walls of sufficient height and thickness were manufactured to carry out tensile tests and to analyze the microstructure.

**Figure 4:**
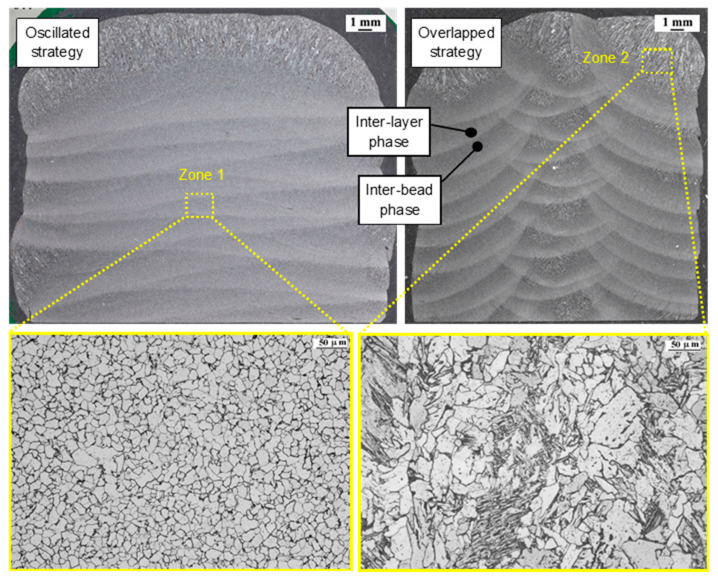
Macrographic images and microstructure of overlapped and oscillated walls manufactured by using GMAW technology with ER70.

Figure 4 shows the typical anisotropy of wire arc additive manufacturing in the arrangement on the inner surface of the wall since the process is produced by the layer-by-layer addition of material. There are clear differences in the layer-by-layer arrangement for each of the strategies (oscillating and overlapping). In the case of oscillation, the presence of a single interface per layer is observed since each layer is formed by a single continuous path. In the case of the overlapping wall, three beads are formed that make up each layer, due to the three paths per individual layer. These interfaces between the weld beads that make up each layer cause there to be less continuity within the wall. As Aldalur et al. [17] demonstrated, the microstructure observed in Zone 1 of Figure 4 is polygonal ferrite since these areas were subjected to thermal cycles. In Zone 2, acicular ferrite appears, as these layers do not experience the thermal effect of the following layer. With the overlapped strategy, a more heterogeneous microstructure is obtained, where polygonal ferrite and acicular ferrite microstructure are intercalated. 

The results of the mechanical tests (UTS, YS and elongation) of the walls in the oscillating and overlapping strategies are summarized in Table 3. UTS, YS and elongation at fracture were calculated from the six samples in each direction engineering stress–strain graph. The YS value is taken at 0.2% engineering strain. The standard deviations in the results of UTS and YS in the vertical direction are smaller than in the horizontal direction. The results are similar to those shown by Rafieazad et al. [18] and slightly lower than those reported by the supplier for this material, as deposited.

As can be seen in Table 3, values of mechanical properties in horizontal and vertical direction are given. In some works from the literature, it is reported that the extraction direction of the specimen has an influence on the mechanical properties. For example, Sun et al. [19] report differences of 10% and 4% in YS and UTS, respectively, and up to 21% in elongation at fracture, assuming that this is related to the density of interfaces between beads, being higher in the vertical test direction. In this work, these differences were not found. Only the elongation was minimally affected by the directionality, being higher in the horizontal direction, which corresponds to that reported by Reference [19]. The presence of a mechanical result independent of the direction of specimen extraction had already been reported in a recent comprehensive study on mechanical properties by Huang et al. [20] wherein the influence of specimen direction, specimen preparation and deposition strategy was studied. In this study, it was concluded that the presence of anisotropy in the mechanical properties of this type of steel is associated with the preparation of the specimen. In machined specimens, the coupons showed almost isotropic mechanical properties, with only some variation in elongation at fracture.

#### 3.1.2. Stainless Steel PAW–WAAM

**Figure 5:**
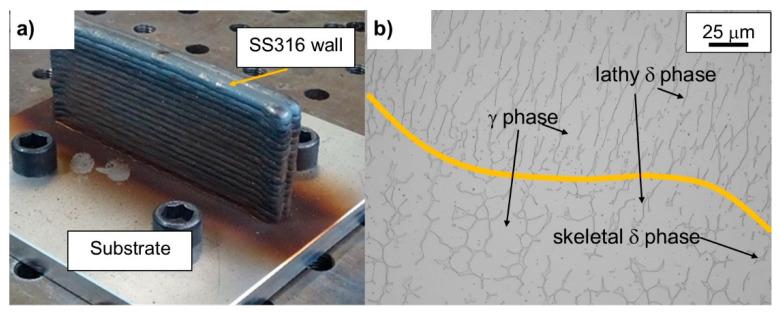
(**a**) Double wall manufactured by PAW with SS 316L-Si. (**b**) Microstructure of the interface zone.

Two identical double walls were fabricated by using the parameters mentioned in Table 4 for PAW technology. The first wall was used to analyze the microstructure of the material in PAW. In this section, the microstructure of a 316L-Si stainless steel alloy fabricated by Plasma Arc Welding (PAW) technology is studied. Figure 5b shows the microstructure of the wall in the union between two weld beads. In the interfacial zone, the concentration of δ-ferrite phases is higher, due to temperature gradients caused by thermal effects. The typical phase structure of ferrite within an austenite matrix is observed. If we focus on the δ-ferrite phase, it is arranged in the form of a skeletal δ phase in the lower part, and a more lathy-like formation in the interface zone. In addition to these structural metallographic characteristics of the SS 316L-Si material manufactured by PAW, no faults such as lack of melting, pores or cracks were observed in any of the processes.

In the second wall made of type 316L stainless steel with two overlapping beads, specimens were extracted for mechanical tests according to both directions already indicated for the previous mild steel. The UTS, YS and fracture elongation results were obtained from four specimens in each direction taken from the wall. Generally, the UTS and YS values indicate better results than the specifications for the as-welded SS 316L, while elongation is slightly lower, but only in the horizontal direction. The results show an anisotropy, the material being stronger but less resilient in the horizontal direction, as reported Suárez et al. [21]. This can be due to the properties of the union, which can affect the properties in that direction.

### 3.2. Bimetallic Wall Manufactured by DED

**Figure 6:**
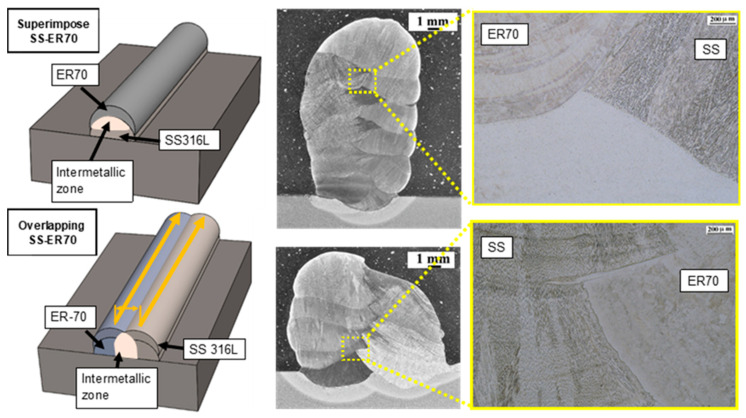
Macrostructure and microstructure of superimposed and overlapped SS-ER70 walls.

In the previous section, we discussed how the properties of each material were tested separately and proved to be optimum. In this section, we discuss the challenge it was to combine both PAW and GMAW technologies to successfully fabricate a bimetallic wall. The walls were manufactured by using two methods (Figure 6): (1) straight wall overlapping beads one upon the other, alternating one bead of each material; and (2) overlapping beads with a separation of 4 mm. 

In both cases, the junction between the two materials, the intermetallic zone, must be analyzed because there is a risk of cracks, pores and underfilling. Figure 6 shows the macroscopic images of both walls. Visually, no cracks or other defects are visible. The intermetallic structure is continuous in both cases. The right side of the image shows the microstructure of the junction, where both parts are visible. As corresponds to each material, ER70S-6 shows a microstructure formed by a bainitic structure, while SS 316L shows δ-ferrite within an austenitic matrix. 

**Figure 7:**
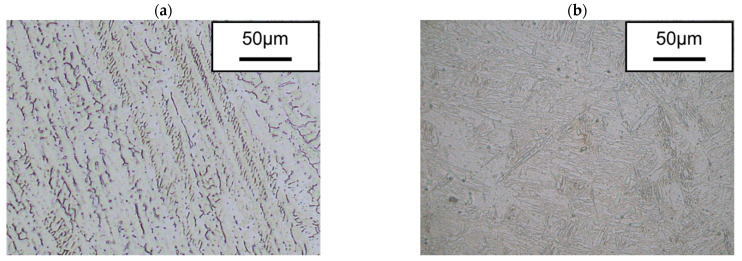
Microstructure of the materials as deposited (**a**) stainless steel SS 316L and (**b**) steel ER70.

Therefore, the intermetallic structure obtained in the fabrication by using WAAM technology to obtain a bimetallic structure of mild steel and stainless steel is continuous and does not show any defects. This demonstrates the potential of this technology to manufacture perfectly functional bimetallic structures without any problems in the junction. Figure 7 shows the microstructures at a higher magnification, where the absence of microcracks is observed. Moreover, Figure 7b shows the formation of molybdenum carbides in the case of ER70.

**Figure 8:**
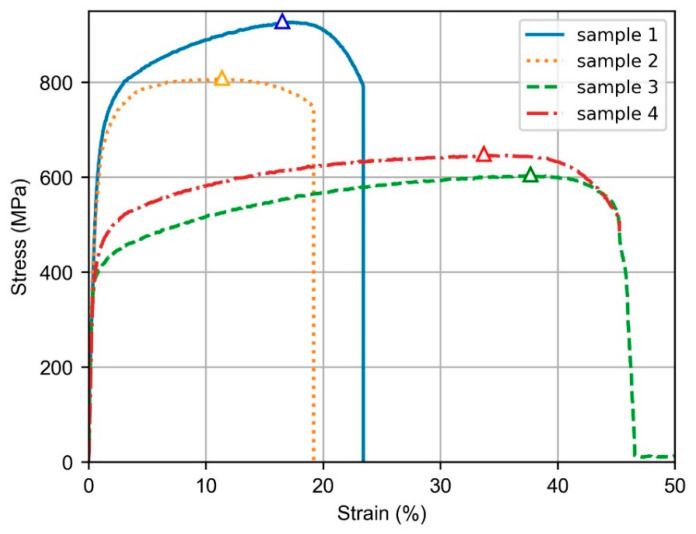
Results of the stress–strain curve for the specimens extracted from the manufactured walls, the triangles show the UTS for each test.

The results of the mechanical characterization tests of the material obtained through the fabrication of bimetallic walls in 316-stainless-steel and ER70-mild-steel structures obtained by means of the two strategies are then analyzed. In Figure 8, the results of the test described are shown for the four specimens taken from Samples 1 and 2 of the superimposed wall and Samples 3 and 4 of the overlapped wall. The average yield strength (YS) values are 584 for Samples 1 and 2 (superimpose wall) and 389 MPa for Samples 3 and 4 (overlapped walls). In the case of the ultimate tensile strength (UTS), 869 MPa is in the superimpose and 628 MPa is in the overlapped. The elongation at break percentage is lower in Samples 3 and 4, with 20%, than in the sandwich, with 36%. However, Samples 1 and 2 are grouped as specimens from the wall manufactured by superimposed strategy. It is observed that Sample 1 obtains higher UTS, YS and elongation values largely due to the presence of the plastic hardening phenomenon.

**Figure 9:**
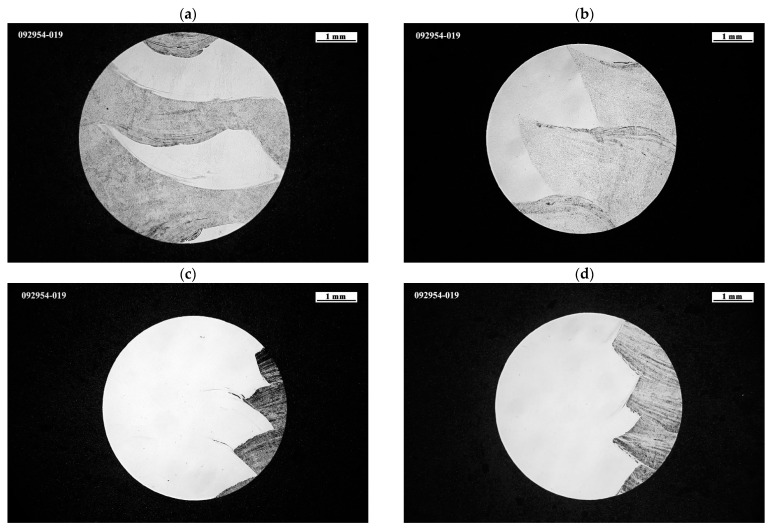
Cross-section of the neck area in the tensile specimens of (**a**) Sample 1, (**b**) Sample 2, (**c**) Sample 3 and (**d**) Sample 4.

The different samples tested were analyzed in order to know the nature of the internal structure; Figure 9 shows the set of specimens tested. The first thing that can be seen from Figure 9a,b is that, although the wall manufacturing strategy is the same—superimposed, in this case—the internal arrangement of the materials in the specimen is different. In the case of Sample 1 Figure 9a, the ER70 and the SS 316L materials are found infiltrated, being mainly the center of the SS3136 specimen, while in Figure 9b, the materials are arranged side by side, as in specimens extracted from the overlapped wall (Figure 9c,d).

**Figure 10:**
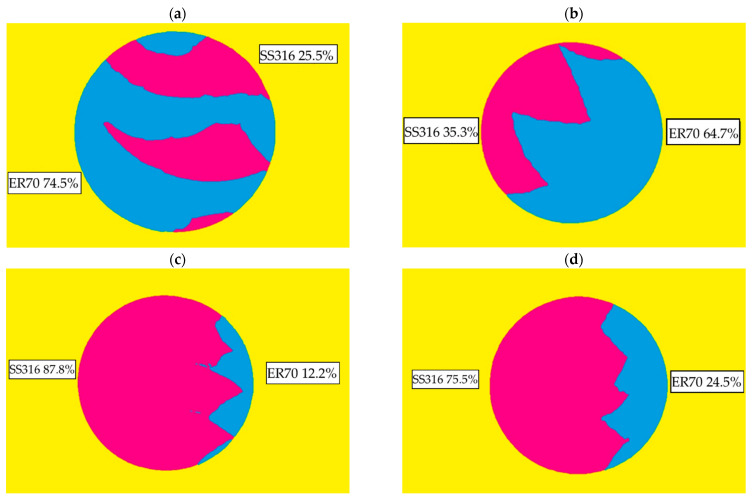
Cross-section material determination in (**a**) Sample 1, (**b**) Sample 2, (**c**) Sample 3 and (**d**) Sample 4.

Taking the structure revealed in Figure 10 and applying a mask, the percentage of each material in the extracted samples can be estimated, as can be seen in Figure 10. The different materials were determined from the macrographs of specimens taken with the Nikon Eclipse MA200 microscope (Tokyo, Japan) and analyzed with Nikon NIS-Elements Imaging Software. The ER70 mild-steel material is colored in blue, while the SS 316L stainless steel is colored in magenta; the color version of Figure 10 can be seen only on the Web version of the paper. It is observed that Sample 1 is the one with the highest percentage of ER70, as seen in Figure 10a. In Samples 3 and 4, on the other hand, the presence of stainless steel is much greater. The nature of the structure and disposition of the metals that form the structure of the specimen in percentage show a high correlation with the mechanical results shown in Figure 7. 

From Figure 10, it can be seen that the specimens, after machining, have a different content of both materials. The random extraction of the specimen, being from the central area of the wall, means that there is not a 50:50 distribution of both materials. This last fact means that a comparison of the superimposing and overlapping strategies cannot be made without taking this factor into account.

In the tensile results obtained (see Figure 8), it can be seen that there is a difference of almost 200 MPa between the UTS observed for Sample 1 and Sample 2. This difference is justified by the presence of ER70 material, with Sample 1 having 10% more, as it can be seen on Figure 10. However, this dependence shows the local mechanical result, and the general behavior of the piece will depend on the percentage distribution of both materials at a more macrolevel. It also allows us to design according to material deposition rates or layer composition; for example, instead of alternate layers of each material, one could have two layers of one material and one layer of the next. This could allow both variable and customized mechanical properties of the wall by optimizing the ratios between the materials to be deposited. For this, a further study on the correlation between mechanical properties and process variables, including the ratio of the two materials and the different deposition strategies, must be performed.

Table 5 shows the Vickers hardness results in different areas of the wall for both technologies. The selected zones are mainly SS 316L in both strategies, and ER70 zones also for superimposed and overlapped. The hardness of the materials does not depend on the strategy used. The quantitative differences do not differ by more than 10 HV in any of the cases. The number of fabricated walls is low; it would be of interest to replicate the study with a larger number of samples. However, within each of the regions, more than six hardness samples were taken so that, for the manufactured walls, the absence of dependence on the strategy was demonstrated. The hardness values in the case of the predominant ER70 zones are high in relation to what has been previously reported in the literature for WAAM material [21]. The increase in hardness is related to a longer waiting time between layers as the set up to deposit the next layer to change of material increases. Moreover, the formation of molybdenum carbides takes place from the diffusion of this component from the stainless steel into the mild steel.

## 4. Conclusions

The present work presents the manufacture of bimetallic walls by using direct energy deposition. Wire arc additive manufacturing (WAAM) technology was selected. Some of the conclusions that can be drawn from this paper are as follows:

- It was proved that bimetallic walls can be manufactured without any considerable defect in the intermetallic zone. No visual defects, no pores and no cracks are observed in the microstructure. Bimetallic walls preserve the microstructure corresponding to each material separately, being interesting in applications where specific properties are required at specific locations.
- The hardness observed in the materials in both strategies is similar, being about 380 HV for ER70 and around 180 HV for SS 316L.
- The specimens from the two strategies were mechanically characterized. It was found that the percentage of steel type in the specimen is decisive. The average values of the ultimate tensile strength (UTS) are 869 and 628 MPa, the yield strength (YS) is 584 and 389 MPa and the elongation at break is 20% and 36%. The former values correspond to the cases with higher % ER70-6, and the latter to those with higher % SS 316. This indicates an important relationship between the distribution of the materials and their mechanical behavior.
- A high incidence of both the deposition strategy and the internal arrangement of the materials in the specimen was observed. The specimens with a higher amount of SS 316L (overlapped walls) show a higher elongation and a lower maximum stress at break.

Finally, as a future line, it should be noted that the DED process is also used to apply coatings that improve the surface characteristics of the resulting parts. With this type of simultaneous manufacturing of bimetallic walls, the coating could also be performed in situ for use in improving corrosion and wear resistance.

## Figures and Tables

**Table 1:** Composition of welding wires used in multi-material parts (wt.%).

	C	Si	Mn	Cr	Ni	Cu	P	Mo	S	Ti	Al	Co	Fe
**ER70S-6**	0.06	0.94	1.64	0.02	0.02	0.02	0.013	0.005	0.016	0.004	-	-	bal.
**SS 316L**	0.007	0.81	1.6	18.26	12.11	0.09	0.019	2.53	0.013	0.003	0.003	0.064	bal.

**Table 2:** Process parameters for additive manufacturing of different materials.

Strategy	Technology	Material	Wire Feed Rate WFR (m/min)	Travel Speed TS (cm/min)	I (A)	V (V)	Overlapping Distance (mm)
Superimposed wall	PAW	SS 316L	3.5	35	250	14.5	-
	GMAW	ER70S-6	3.5	35	120	23	
Overlapped wall	PAW	SS 316L	3.5	35	250	14.5	4
	GMAW	ER70S-6	3.5	45	120	23	

**Table 3:** Summary comparison of mechanical characterization on GMAW-ER70 wall on different directions.

		Tensile Test		
		UTS (MPa)	YS 0.2% (MPa)	Elong. (%)
**ER70 WAAM**	Horizontal	498 ± 9	368 ± 12	36 ± 4
	Vertical	501 ± 3	368 ± 4	32 ± 1
**ER70 as welded**	-	500–640	>420	28

**Table 4:** Summary comparison of mechanical characterization on PAW-SS 316 wall in different directions.

		Tensile Test		
		UTS (MPa)	YS 0.2% (MPa)	Elong. (%)
**SS 316L WAAM**	Horizontal	581 ± 10	381 ± 15	38 ± 4
	Vertical	534 ± 9	341 ± 6	52 ± 11
**SS 316L as welded**	-	>525	>220	45

**Table 5:** Summary comparison of hardness characterization on SS 316L side and ER70 side on walls fabricated under different strategies.

	Hardness Test (HV)			
	Superimposed Wall		Overlapped Wall	
	Mean	SD	Mean	SD
**SS 316L WAAM**	178	9	186	10
**ER70 WAAM**	385	16	379	7

## Data Availability

Not applicable.
